# Postpartum Sacral Stress Fracture in a Primiparous Woman: A Case Report

**DOI:** 10.7759/cureus.100970

**Published:** 2026-01-07

**Authors:** Hilal Caglar, Serdar Hira, Sabri Onur Caglar

**Affiliations:** 1 Physical Medicine and Rehabilitation, Royal Hospital Bandırma, Balıkesir, TUR; 2 Clinical Biochemistry, Royal Hospital Bandırma, Balıkesir, TUR; 3 Cardiology, Private Royal Hospital, Balıkesir, TUR

**Keywords:** case report, low back pain, mri - magnetic resonance imaging, pregnancy and the postpartum period, sacral stress fracture

## Abstract

Sacral stress fractures are rare in young adults and are an uncommon cause of postpartum lumbosacral pain. Due to nonspecific symptoms and frequently normal initial radiographs, diagnosis is often delayed. Increased mechanical loading and pregnancy-related biomechanical changes are thought to contribute to their development. We report the case of a 32-year-old primiparous woman who presented with progressive low back and unilateral buttock pain beginning 18 days after an uncomplicated vaginal delivery. Physical examination revealed focal sacral tenderness and pain with provocative sacroiliac maneuvers. Laboratory investigations were unremarkable except for vitamin D insufficiency. Plain radiographs were normal. Magnetic resonance imaging (MRI) demonstrated a unilateral stress fracture of the left sacral ala with associated bone marrow edema. Bone mineral density assessment showed normal values, supporting a fatigue-type fracture. The patient was treated conservatively with activity modification, analgesia, and calcium-vitamin D supplementation. Complete symptom resolution was achieved within 10 weeks. Postpartum sacral stress fractures should be considered in women presenting with focal sacral pain that worsens with weight-bearing, even in the absence of metabolic bone disease. Early MRI facilitates prompt diagnosis and prevents unnecessary diagnostic delays. Conservative management is usually effective and results in excellent clinical outcomes.

## Introduction

Sacral stress fractures are an uncommon cause of lumbosacral pain and are rarely encountered in young adults, particularly during the postpartum period [1]. Owing to their nonspecific clinical presentation, these fractures are frequently misdiagnosed as more common postpartum musculoskeletal conditions such as sacroiliac joint dysfunction or lumbar spine pathology, resulting in delayed diagnosis and prolonged symptoms [2]. Patients typically present with low back, buttock, or pelvic pain that worsens with weight-bearing activities and improves with rest [2].

These fractures may develop either as fatigue-type injuries caused by repetitive mechanical loading of normal bone or as insufficiency fractures resulting from physiological stress on structurally weakened bone [3]. Pregnancy-related biomechanical and metabolic changes, including increased lumbar lordosis, gestational weight gain, hormonal ligamentous laxity, and increased skeletal metabolic demands, may contribute to their development [4].

Because the clinical presentation often overlaps with more common postpartum conditions, diagnosis is frequently delayed. Conventional radiographs are often unremarkable in the early stages, whereas magnetic resonance imaging (MRI) is the preferred modality for detecting bone marrow edema and subtle fracture-related changes.

Here, we present the case of a 32-year-old primiparous woman who developed a unilateral sacral stress fracture shortly after an uncomplicated vaginal delivery, highlighting the diagnostic challenges and the importance of early MRI in postpartum patients with focal sacral pain.

## Case presentation

A 32-year-old primiparous woman presented with progressively worsening low back and left buttock pain that began 18 days after an uncomplicated spontaneous vaginal delivery of a healthy 2900 g infant. She had gained 11 kg during pregnancy and was exclusively breastfeeding at the time of presentation. The pain was described as a deep, aching sensation localized to the left sacral region. It intensified during weight-bearing activities such as walking, stair climbing, or carrying her infant, but improved substantially with rest. She denied nocturnal pain, trauma, strenuous exercise, or prior episodes of similar discomfort. Her medical history was unremarkable, and she had no known risk factors for osteoporosis.

Physical examination revealed pronounced tenderness over the left sacroiliac area. Provocative maneuvers, including the FABER and Gaenslen tests, reproduced her symptoms, while the neurological examination of both lower limbs was normal. An antalgic gait was observed due to pain during left-sided weight-bearing. Pain intensity was assessed clinically using the Visual Analog Scale (VAS). At initial presentation, the patient reported a VAS score of 7/10, indicating moderate to severe pain predominantly during weight-bearing activities. No formal functional disability questionnaire or proprietary scoring system was applied, given the acute presentation and rapid clinical improvement.

Laboratory tests, including serum calcium, phosphate, renal function, thyroid function, and parathyroid hormone, were within normal limits (Table 1). The 25-hydroxyvitamin D level was 10.4 ng/mL, which was consistent with vitamin D insufficiency.

**Table 1:** Laboratory values at administration with normal ranges ALT: Alanine aminotransferase, AST: Aspartate aminotransferase, ALP: Alkaline phosphatase, LDH: Lactate dehydrogenase, CRP: C-reactive protein, T4: Thyroxine, TSH: Thyroid-stimulating hormone

Test	At administration	Reference ranges
Complete blood count		
Hemoglobin (g/L)	13.6	11.6-15
Hematocrit	39.4	36-44
Leukocytes (10^9^/L)	5.78	4.5-11
Thrombocytes (10^9^/L)	295	150-450
Biochemistry		
Glucose (mg/dL)	95.9	70-99
Sodium (mmol/L)	139	135-145
Potassium (mmol/L)	4.74	3.6-5.2
Chloride (mmol/L)	100	96-106
Calcium (mg/dL)	10.4	8.6-10.3
Phospate (mg/dL)	3.85	2.5-4.5
Magnesium (mg/dL)	1.98	1.7-2.2
Creatinine (mg/dL)	1.075	0.6-1.2
Albumin (g/dL)	4.51	3.5-5.5
ALT (U/L)	16.3	7-55
AST (U/L)	17	10-40
ALP (U/L)	78	35-104
LDH (U/L)	146	135-214
Creatine kinase (U/L)	71	38-308
CRP (mg/L)	3.53	<5
Erythrocyte sedimentation rate (1 hour)	14	8-20
Free T4 (ng/dL)	1.19	0.93-1.77
TSH (uIU/mL)	2.11	0.27-4.2
Vitamin B12 (pg/mL)	591.7	191-946
Parathormone-intact (pg/mL)	21.3	15-65
Vitamin D (ng/mL)	10.4	30-100

**Figure 1:**
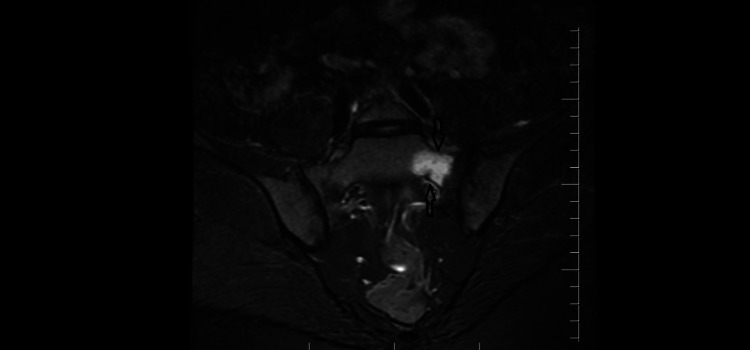
Coronal short tau inversion recovery (STIR) magnetic resonance imaging (MRI) of the pelvis The image shows marked bone marrow edema involving the left sacral ala (arrow), consistent with an acute sacral stress fracture in the postpartum period

Lumbosacral and pelvic radiographs did not reveal any abnormalities. Given persistent focal pain, an MRI of the pelvis and sacrum was performed. The MRI demonstrated a distinct linear low-signal fracture line through the left sacral ala, accompanied by extensive bone marrow edema on short tau inversion recovery (STIR) sequences, consistent with an acute sacral stress fracture (Figure 1). No soft tissue abnormalities were identified.

To exclude metabolic bone disease, a dual-energy X-ray absorptiometry (DEXA) scan was performed four weeks after diagnosis, showing a lumbar spine Z-score of −0.4, left femoral neck Z-score of 1.1, and left femoral total T score of 0.6. These findings indicated normal bone density, suggesting that the fracture represented a fatigue-type mechanism rather than an insufficiency fracture.

The patient was managed conservatively. She was advised to limit weight-bearing for two weeks and gradually resume normal ambulation as tolerated. No assistive walking device was prescribed during management, as the patient was able to ambulate independently without instability, neurological deficit, or significant gait disturbance at presentation. The decision was based on preserved weight-bearing capacity, normal neurological examination, stable balance, and pain that was activity-related but tolerable at rest. Analgesics and non-steroidal anti-inflammatory drugs were prescribed as needed for pain control. Calcium and vitamin D supplementation was initiated using a combined preparation providing elemental calcium equivalent to 1000 mg (from 2500 mg calcium carbonate) and vitamin D3 equivalent to 880 IU, to support bone health during lactation. Heavy lifting and prolonged standing were discouraged. 

By the fourth week of conservative treatment, the patient’s symptoms had significantly improved. During follow-up, pain intensity progressively decreased with conservative management, and the VAS score declined to 2/10 by 10 weeks postpartum, corresponding with complete functional recovery and return to daily activities. At 10 weeks postpartum, she remained asymptomatic. Repeat imaging was not deemed necessary due to full symptomatic recovery.

## Discussion

Sacral stress fractures occurring during the postpartum period are uncommon and frequently underdiagnosed due to their nonspecific clinical presentation and significant overlap with more prevalent causes of postpartum lumbosacral pain [2]. Conditions such as sacroiliac joint dysfunction, lumbar disc disease, and muscular strain are far more commonly considered in this setting, often leading to delayed recognition of stress-related sacral pathology. In the present case, the presence of focal unilateral sacral pain that was exacerbated by weight-bearing activities and relieved by rest prompted further diagnostic evaluation beyond routine postpartum musculoskeletal explanations.

The underlying pathophysiology of postpartum sacral stress fractures is not fully elucidated, and two principal mechanisms have been proposed. Fatigue-type fractures may develop as a result of repetitive mechanical loading applied to structurally normal bone, whereas insufficiency fractures occur in the context of reduced bone strength due to metabolic or hormonal factors [3,5]. Pregnancy-related biomechanical adaptations, including increased lumbar lordosis, gestational weight gain, altered gait mechanics, and repetitive physical demands associated with infant care, may increase stress across the sacral ala and predispose susceptible individuals to fatigue-type fractures [4]. In contrast, pregnancy- or lactation-associated osteoporosis has been described in a subset of postpartum patients and may contribute to insufficiency-type fractures [5,6].

In this patient, a systematic evaluation was undertaken to distinguish between these potential mechanisms and to exclude alternative etiologies. Laboratory investigations assessing calcium-phosphate metabolism, thyroid and parathyroid function, and inflammatory markers were within normal limits, except for vitamin D insufficiency. DEXA demonstrated normal bone mineral density, effectively excluding pregnancy-associated osteoporosis and other forms of metabolic bone disease. In addition, there were no recognized obstetric risk factors such as fetal macrosomia, prolonged labor, or instrumental delivery. Collectively, these findings favored a fatigue-type stress fracture mechanism rather than an insufficiency fracture.

Several alternative diagnoses were also considered and carefully excluded. Sacroiliitis was deemed unlikely due to the absence of inflammatory back pain characteristics, normal inflammatory markers, and preserved sacroiliac joint architecture on MRI. Lumbar disc herniation and degenerative spinal disease were excluded based on the lack of neurological deficits and the poor correlation between incidental lumbar imaging findings and the patient’s focal sacral pain pattern. Infectious causes were considered improbable given the absence of fever, systemic symptoms, or laboratory evidence of infection. This structured diagnostic approach supported the diagnosis of a mechanical stress-related sacral fracture rather than inflammatory, infectious, or degenerative conditions.

MRI played a decisive role in establishing the diagnosis. While plain radiographs are frequently normal in the early stages of stress fractures, MRI allows sensitive detection of bone marrow edema and subtle fracture lines [1,2]. In this case, MRI revealed a linear fracture line through the left sacral ala accompanied by extensive marrow edema, findings characteristic of an acute sacral stress fracture. These imaging features are consistent with previously reported postpartum cases and reinforce MRI as the diagnostic modality of choice when sacral stress fracture is suspected in postpartum patients with persistent or localized pain [1,2,5,7].

Management of postpartum sacral stress fractures is typically conservative and focuses on activity modification, pain control, and optimization of bone health [2]. Our patient was advised to temporarily limit weight-bearing and received analgesics as needed. Given the documented vitamin D insufficiency and ongoing lactation, calcium and vitamin D supplementation were initiated using a combined preparation to support skeletal recovery. The patient demonstrated progressive clinical improvement and achieved complete symptom resolution within 10 weeks, consistent with outcomes reported in the literature.

## Conclusions

This case highlights the importance of maintaining a high index of suspicion for sacral stress fractures in postpartum women presenting with focal sacral or buttock pain that worsens with mechanical loading, even in the absence of metabolic bone disease or obstetric risk factors. Early use of MRI is crucial for establishing an accurate diagnosis when initial radiographs are normal and clinical findings are nonspecific. Prompt recognition enables appropriate conservative management, prevents unnecessary diagnostic delays, and is associated with excellent clinical outcomes.

## References

[1] Gul Z (2025). Case report: postpartum sacral stress fracture. Eur J Med Sci.

[2] Wu YF, Lu K, Girgis C, Preda M, Preda V (2021). Postpartum bilateral sacral stress fracture without osteoporosis-a case report and literature review. Osteoporos Int.

[3] Akhaddar A (2023). Sacral stress fractures. Atlas of Sciatica: Etiologies, Diagnosis.

[4] Yoseph ET, Taiwo R, Kiapour A (2025). Pregnancy-related spinal biomechanics: a review of low back pain and degenerative spine disease. Bioengineering (Basel).

[5] Hilal N, Nassar AH (2016). Postpartum sacral stress fracture: a case report. BMC Pregnancy Childbirth.

[6] Yan CX, Vautour L, Martin MH (2016). Postpartum sacral insufficiency fractures. Skeletal Radiol.

[7] Erdem MN, Kültür Y, Akar A, Aydoğan M (2025). A rare cause of postpartum lower back pain: sacrum stress fractures. J Turk Spinal Surg.

